# Draft Genome Sequence of an Anthracimycin Producer, *Streptomyces* sp. TP-A0875

**DOI:** 10.1128/genomeA.01149-15

**Published:** 2015-10-08

**Authors:** Hisayuki Komaki, Natsuko Ichikawa, Akira Hosoyama, Nobuyuki Fujita, Enjuro Harunari, Yasuhiro Igarashi

**Affiliations:** a Biological Resource Center, National Institute of Technology and Evaluation (NBRC), Chiba, Japan; b NBRC, Tokyo, Japan; c Biotechnology Research Center and Department of Biotechnology, Toyama Prefectural University, Toyama, Japan

## Abstract

Here, we report the draft genome sequence of an anthracimycin producer, *Streptomyces* sp. TP-A0875. The genome contains at least two type I polyketide synthase (PKS) gene clusters, two type II PKS gene clusters, and three nonribosomal peptide synthetase gene clusters. The gene cluster for anthracimycin biosynthesis was identified based on the PKS domain organization.

## GENOME ANNOUNCEMENT

In our screening for inhibitors of tumor cell invasion, *Streptomyces* sp. TP-A0875 isolated from a compost sample was found to produce a tricyclic macrolide which was later designated anthracimycin (1, 2). The polyketide backbone of anthracimycin is identical with that of chlorotonil A produced by a myxobacterium (3). It is quite unusual that the compounds bearing the same carbon skeleton are produced by different organisms phylogenetically apart. To identify the genes for anthracimycin biosynthesis, the genome of strain TP-A0875 was sequenced.

*Streptomyces* sp. TP-A0875 was deposited at the NBRC culture collection (NBRC 110026). The whole genome of *Streptomyces* sp. TP-A0875 monoisolate was read by using a combined strategy of shotgun sequencing with GS FLX+ (Roche; 53.2 Mb sequences, 7.9-fold coverage) and pair-end sequencing with MiSeq (Illumina; 665.8 Mb, 98-fold coverage). These reads were assembled using Newbler v2.6 software, and subsequently finished using GenoFinisher software (4), which led to a final assembly of 39 scaffold sequences of >500 bp each. The total size of the assembly was 6,778,367 bp, with a G+C content of 73.6%. Coding sequences were predicted by Prodigal (5), and surveyed for polyketide synthase (PKS) and nonribosomal peptide synthetase (NRPS) gene clusters as previously reported (6).

The genome contained at least two type I PKS, two type II PKS, and three NRPS gene clusters. One of the type I PKS gene clusters, encoded in Scaffold04, contained a discrete acyltransferase (AT) (orf93) and three modular PKS genes (orf92 to orf90) lacking AT domains. Based on the domain organization of PKSs, these genes were deduced to be responsible for anthracimycin biosynthesis. Another type I PKS gene cluster was divided into a couple of scaffolds (Scaffold07, Scaffold32, Scaffold34), but high sequence similarities (>68%) to macrolactam PKSs such as HitP (7), Mla (8), Bec (9), and CmiP (10) suggested that this PKS cluster is involved in macrolactam production. The type II PKS gene cluster, encoded in Scaffold03, is likely responsible for rubromycin biosynthesis because its ketosynthases, KSα (orf98) and KSβ (orf97), and acyl carrier protein (ACP, orf96) showed high sequence similarities (93 to 96%) to RubA, RubB, and RubC, respectively (11). Another type II PKS coded in Scaffold06 likely synthesizes a spore pigment since its KSα (orf17), KSβ (orf16), and ACP (orf15) showed 93 to 95% sequence similarities to WhiE proteins (12). An NRPS gene cluster in Scaffold04 is proposed to produce a siderophore comprising ornithine/threonine/ornithine by analyzing with antiSMASH (13) and BLAST searches. An NRPS gene cluster in Scaffold14 would be responsible for tetrapeptide including threonine and valine. It was unable to predict the structure of a peptide from an NRPS gene cluster in Scaffold07 due to the incompleteness of the single module (the cluster contained only a single module composed of adenylation-thiolation-thioesterase domains). The gene cluster for anthracimycin biosynthesis is present also in *Streptomyces* sp. NRRL F-5065 (GenBank accession no. JOHV00000000.1).

### Nucleotide sequence accession numbers.

The draft genome sequence of *Streptomyces* sp. TP-A0875 has been deposited in the DDBJ/ENA/GenBank database under the accession no. BBZE00000000. The version described in this paper is the first version, BBZE01000000.
